# Effect of Dietary Combination of Methionine and Fish Oil on Cellular Immunity and Plasma Fatty Acids in Infectious Bursal Disease Challenged Chickens

**DOI:** 10.1155/2013/531397

**Published:** 2013-10-01

**Authors:** Elham Maroufyan, Azhar Kasim, Goh Yong Meng, Mahdi Ebrahimi, Loh Teck Chwen, Parvaneh Mehrbod, Behnam Kamalidehghan, Abdoreza Soleimani Farjam

**Affiliations:** ^1^Department of Animal Science, Universiti Putra Malaysia, 43400 Serdang, Selangor, Malaysia; ^2^Department of Preclinical Sciences, Universiti Putra Malaysia, 43400 Serdang, Selangor, Malaysia; ^3^Institute of Bioscience, Universiti Putra Malaysia, 43400 Serdang, Selangor, Malaysia; ^4^Pharmacy Department, Faculty of Medicine, University of Malaya, Malaysia; ^5^Institute of Tropical Agriculture, Universiti Putra Malaysia, 43400 Serdang, Selangor, Malaysia

## Abstract

This study was carried out to investigate the modulatory effects of dietary methionine and fish oil on immune response, plasma fatty acid profile, and blood parameters of infectious bursal disease (IBD) challenged broiler chickens. A total of 300 one-day-old male broiler chicks were assigned to one of six dietary treatment groups in a 3 × 2 factorial arrangement. There were three levels of fish oil (0, 2.5 and 5.5%), and two levels of methionine (NRC recommendation and twice NRC recommendation). The results showed that the birds fed with 5.5% fish oil had higher total protein, white blood cell count, and IL-2 concentration than those of other groups at 7 days after IBD challenge. Inclusion of fish oil in diet had no effect on IFN-**γ** concentration. However, supplementation of methionine twice the recommendation enhanced the serum IFN-**γ** and globulin concentration. Neither of fish oil nor methionine supplementation affected the liver enzymes concentration. It can be suggested that a balance of moderate level of fish oil (2.5%) and methionine level (twice NRC recommendation) might enhance immune response in IBD challenged broiler chickens.

## 1. Introduction

The strategies to enhance immune-functional abilities through nutrition have extended to poultry nutrition in the last decade. Today, polyunsaturated fatty acids (PUFA) are widely accepted as a part of modern nutrition, because of their beneficial health-promoting effect in animal and human diets [1, 2]. Several empirical studies have shown that modification of dietary fatty acids alters the fatty acid composition of tissue lipids [3–5] and has notable effect on the inflammatory immune response [2, 6–9]. These modulations are regulated through bioactive lipid mediators such as eicosanoids, prostaglandins, leukotrienes, lipoxins, and resolvins [10]. Interestingly, it has been reported that *n-3* PUFA intake may attenuate the growth-inhibitory effects of proinflammatory cytokine in various species [9, 11–13]. Moreover, inclusion of fish oil as a source of precursors for eicosanoids in the diet appears to improve humoral immunity and ameliorate the suppression of the cellular immune response caused by prostaglandin E2 (PGE2) [14, 15]. 

On the other hand, methionine as an essential amino acid is linked to PUFA metabolism. The methionine-homocysteine cycle produces methyl groups for the synthesis of phosphatidylcholine from phosphatidylethanolamine [16, 17]. Phosphatidylcholine is essential for the delivery of PUFA from the liver to the plasma and tissues. In an early study, Tidwell [18] found that fat absorption increased when lipotropic amino acid such as methionine was ingested along with the lipids. It has also been shown that high methionine supplementation (224 mg/kg body weight) increases docosahexaenoic acid in the liver and jejunum [19]. Meanwhile, S-adenosylmethionine, a product of methionine metabolism, plays an important role as the methyl group donor in transmethylation reactions, in which the synthesis of membrane phospholipids (particularly phosphatidylcholine) is necessary for the maintenance of membrane fluidity [20]. Accordingly, it is reasonable to expect a more effective dietary manipulation response when methionine and PUFA are supplemented together. An immune challenge approach may reveal this interaction effect more clearly. Therefore, infectious bursal disease (IBD) as a common disease in poultry industry is used in our study to investigate this hypothesis by feeding different dietary combination of fish oil and methionine to broiler chickens.

## 2. Materials and Methods

### 2.1. Birds and Housing

A total of 300 one-day-old male broiler chicks (Cobb) were purchased from a local hatchery. The chicks were individually wing-banded, weighed, and housed in cages in the open sided house with cyclic temperature (minimum, 24°C; maximum, 34°C). The relative humidity was between 80 and 90%. Feed and water were provided *ad libitum* and lighting was continuous.

### 2.2. Experimental Design

Experimental procedure was approved by the (ACUC) Animal Care and Use Committee of Universiti Putra Malaysia. Commencing from day one, five replicate cages of 10 chicks each were assigned to one of the six dietary treatments, giving a total of 30 pens. The diets were formulated to meet or exceed the requirements of the National Research Council (NRC, 1994) for broilers of this age [21]. There were three levels of tuna oil (0, 2.5, and 5.5%) and two levels of DL-methionine (NRC recommendation and twice NRC recommendation). Therefore, the following six dietary treatments were compared: (1) basal diet based on NRC recommendation (M_1_F_0_); (2) basal diet containing methionine 2 times higher than NRC (M_2_F_0_); (3) basal diet containing 2.5% tuna oil + 3.5% sunflower oil (M_1_F_2.5_); (4) basal diet containing 5.5% tuna oil + 0.5% sunflower oil (M_1_F_5.5_); (5) combination of diets 2 and 3 (M_2_F_2.5_); (6) combination of diets 2 and 4 (M_2_F_5.5_) (Tables 1 and 2). The choice of tuna oil in our study was based on the commercial availability of oil in large scale and the higher level of docosahexaenoic acid (DHA) compared with other fish oil sources. To prevent lipid peroxidation, precautions were taken by mixing feed every two weeks and addition of butylated hydroxytoluene (BHT) and ethoxyquin (EQ) as antioxidants (100 g/ton) to diets. 

### 2.3. Chemical Analysis

The proximate chemical analysis of the feeds was carried out following standard methods of AOAC (2000) [22]. The dry matter was determined by oven drying in a forced-air oven for 24 h at 105°C. The Kjeltec Auto Analyzer (Tecator, Hoganas, Sweden) was used to determine nitrogen and then converted to crude protein (CP = *N* × 6.25), while the ether extract (EE) was determined in petroleum ether using a 2025 Soxtec Auto Analyzer (Tecator, Hoganas, Sweden). The ash content was determined by ashing the samples in a muffle furnace at 550°C for 4 h.

### 2.4. Amino Acid Composition of Diets

Amino acids were analyzed by hydrolyzing samples (0.2 g) with 5 mL of 6 N HCl at 110°C for 24 hours in sealed evacuated tubes to obtain hydrolyzate suitable for analyzing all amino acids except methionine and cysteine [23]. An internal standard was then added into the cooled hydrolyzate which was diluted with deionized water as well as 10 *μ*L of this filtrate as mixed with 70 *μ*L of AccQ-Fluor borate buffer and 20 *μ*L of AccQ-Fluor reagent. Then the samples were analyzed by using high-performance liquid chromatography (HPLC) with a Waters 717 Plus HPLC autosampler and a Waters 2475 multi-*λ* fluorescence detector set at an excitation wavelength of 250 nm and an emission wavelength of 395 nm. Separation was achieved in a Waters AccQ-Tag amino acid analysis column, 3.9 × 150 mm at a flow rate of 1 mL/min (Waters Corporation, Milford, MA, USA). Cystine and methionine were analyzed as cysteic acid (Cya) and methionine sulphone (MetO_2_), respectively, by oxidation with performic acid for 16 h at 4°C and neutralization with hydrobromic acid before hydrolysis. 

### 2.5. Challenge Protocol

The clinical form of IBD usually occurs in chickens from 3 to 6 weeks of age. Thus, on day 28 of age, all birds were challenged orally with commercial live IBD vaccine (V877 strain, Malaysian Vaccines and Pharmaceuticals Sdn. Bhd). The live vaccine was chosen to induce infection in the birds. The strain was characterized as an intermediate classical strain which is used under normal conditions as a standard procedure applying for most situations in the field. Each bird was inoculated with a dose of 10^4.0^ EID_50_ IBD viruses into the lumen of the crop by oral gavage [24].

### 2.6. Fatty Acid Analysis

The total fatty acids were extracted from diets and plasma samples using chloroform: methanol 2 : 1 (v/v) based on the method by Folch et al. [25] and modified by Ebrahimi et al. [26] with an addition of antioxidant (0.2 mg/L BHT) to prevent oxidation during sample preparation. The experimental diets and plasma were mixed in 40 mL chloroform : methanol (2 : 1 v/v). Transmethylation of the extracted fat to fatty acid methyl esters (FAME) were carried out using KOH in methanol and 14% methanolic boron trifluoride (BF_3_) (Sigma Chemical Co. St. Louis, Missouri, USA) according to the methods in AOAC (2000). The methyl esters were quantified by gas chromatography (Agilent 7890A) using a 30 m × 0.25 mm ID (0.20 *µ*m film thickness) Supelco SP-2330 capillary column (Supelco, Inc., Bellefonte, PA, USA). One microliter of FAME was injected by an autosampler into the chromatograph, equipped with a split/splitless injector and a flame ionization detector (FID). The injector temperature was programmed at 250°C, and the detector temperature was 300°C. The column temperature program was initiated to run at 100°C, for 2 min, warmed to 170°C at 10°C/min, held for 2 min, warmed to 220°C at 7.5°C/min, and then held for 20 min to facilitate optimal separation. All results of fatty acid were presented as the percentage of total fatty acids. All peaks were quantified using fatty acid standards (Supelco 18919, fatty acid methyl ester mixture, USA).

### 2.7. Serum Chemistry and Total White Blood Cell Count

On day 28 ( before challenge), 35 (7 days after challenge), and 42 (14 days after challenge), five birds from each treatment groups were randomly chosen and their blood samples (3.0 mL) were collected from the brachial vein using a 23-gauge needle. Five different birds were used each time for sampling. The blood samples were immediately aliquoted into non-anticoagulant and anticoagulant tubes containing K-EDTA as an anticoagulant. Blood in the nonanticoagulant tubes was allowed to clot for 2 h at 37°C, and then the serum was decanted [27]. The blood samples in the anticoagulant tubes were packed on ice until they were centrifuged (3000 g for 15 min). Serum and plasma were stored at −20°C until analysis. Serum total protein, albumin, globulin, alanine aminotransferase (ALT), aspartate aminotransferase (AST), cholesterol, and triglyceride were measured by specific commercial kits (Roche Diagnostica, Basel, Switzerland) using an autoanalyzer (Hitachi 902 automatic auto analyzer). Total WBC counts were determined using an automated hematological analyzer (Cell-Dyn 3700; Abbott Laboratories, Abbott Park, IL, USA).

The IL-2 and IFN-*γ* levels in the serum were measured using chicken ELISA kit (Cusabio Biotech, CA, USA) and microplate reader (Bio-Tek Instruments Inc. ELX 800; Winooski, VT) [28, 29] according to the manufacturer's recommendation.

### 2.8. Statistical Analysis

Data were analyzed using the GLM procedure of SAS [30]. Data were subjected to 2-way ANOVA in a 3 × 2 factorial arrangement with fish oil and DL-methionine as the main effects and their interactions. When interactions were significant, a separate ANOVA was conducted within each main effect. Significant differences were separated using Duncan's multiple range tests. The results were expressed as mean ± SEM. Statistical significance was considered at *P* < 0.05.

## 3. Results

Fatty acid composition analysis of plasma showed that there is no significant interaction between dietary methionine and fish oil (Table 3). However, supplementation of fish oil increased plasma *n-3* PUFA level (*P* < 0.05) and decreased *n-6/n-3 *compared to the control group. Methionine supplementation twice the recommended level was not affected the plasma fatty acids profile (*P* > 0.05).

No significant interaction was observed for total white blood cell, plasma total protein, albumin, and globulin throughout the study (Table 4). Before challenge and 7 days after challenge, birds of F_5.5_ group had significantly higher total WBC than F_2.5_ and F_0_ birds. These birds had also higher total protein at before challenge period and higher total protein at 7 days after challenge than the other two groups (*P* < 0.05). At 7 days after challenge, the concentration of globulin was significantly higher in M_2_ group than M_1_. At 14 days after challenge, there were no differences between treatment groups for all the parameters measured in this study, and it seems that the birds were fully recovered from the IBD challenge by this time. In addition, the concentration of liver enzymes, cholesterol, and triglyceride in serum was not influenced by methionine or fish oil supplementation in both prechallenge and 14 days postchallenge periods (Tables 5 and 6).

The effects of fish oil and methionine supplementation on serum IL-2 and IFN-*γ* are shown in Table 7. Regardless of methionine supplementation, the concentration of IL-2 was higher (*P* < 0.05) in F_5.5_ birds compared to F_0_ and F_2.5_ at 7 days after challenge. There were significant interactions between dietary fish oil and methionine for IL-2 at 2 days after challenge and IFN-*γ* at 7 days after challenge. Comparison of the interaction effect was revealed that only the birds of M_2_ group which were supplemented with fish oil had lower serum IL-2 at 2 days after challenge (*P* < 0.05) (Table 8). However, methionine supplementation at twice the recommendation was increased IFN-*γ* concentration only in birds with no fish oil supplementation (M_2_F_0_). On the other hand, these groups of birds (F_2.5_ and F_5.5_) and showed lower concentration of IFN-*γ* only when they were supplemented with twice the methionine recommendation.

## 4. Discussion

Dietary *n-3* PUFA enrichment alters the fatty acid profile of plasma and meat towards higher level of long chain PUFA [31–33]. In agreement, as indicated by current study results, the total *n-3 *PUFA, EPA, and DHA of plasma significantly increased with inclusion of fish oil in diet. In addition, our results are consistent with Khalifa et al. [34] showing that the dietary *n-3* PUFA enrichment decreases the proportion of arachidonic acid (C20:4 *n-6*) in chicken plasma. It has been shown that the fatty acid composition of phospholipid fraction of plasma is closely related to the fatty acid composition of erythrocyte and platelet membrane phospholipids [35]. Therefore, plasma phospholipid fatty acids have the potential to function as a surrogate measure of the potential effects of diet on the whole range of cell membrane lipids. This noninvasive measure may facilitate the short or long term dietary fatty acid modulation studies in the chicken model. The addition of fish oil also may improve the absorption of fat-soluble vitamins, decrease pulverulence, increase palatability, reduce the rate of food passage, and allow a better absorption of all nutrients present in the diet [36]. The dietary supplementation of fish oil increased the number of WBC in the peripheral blood, indicating an immune-stimulatory effect of *n-3 *essential fatty acids. This finding coincides with the report of Mansoub [37] that feeding high *n-3* diet increased WBC count, total protein, and globulin.

Regarding the immunological challenge of the study, an activation of immune system was observed, as indicated by the lower serum IL-2 and higher IFN-*γ* concentration at 2 days after challenge compared to before challenge condition. The *n-3* fatty acids and fish oil are generally known to decrease the levels of proinflammatory cytokines such as IL-1, IL-2, IL-6, and TNF-*α* [38–40]. It has been reported that *n-3 *PUFA supplementation increased T-cell proliferation and enhanced IL-2 production by splenocytes in mice [41]. It has also shown that PUFA deficiency may reduce the lymphocyte proliferation, IL-2 production, monocyte, and polymorphonuclear cell chemotaxis in mammals [34, 42, 43]. Consistently, Sijben et al. [15] showed that IL-2 expression enhanced in lipopolysaccharide- (LPS-) injected birds fed fish oil rich diet [44]. Similarly, in our study, supplementation of fish oil enhanced IL-2 response and suppressed IFN-*γ* level. This immune-modulating effects from feeding diets rich in *n-3* PUFA may be explained by the capacity of the *n-3* PUFA to reduce prostaglandin E (PGE) production through competition with arachidonic acid as a substrate for cyclooxygenase [10]. In infections, reduction of PGE stimulates immunity by increasing TNF [45] and IL-2 [2]. However, the reduction of IFN-*γ* level and consequently inflammation and immune response in fish oil supplemented birds are not clear and may not be explained by this mechanism. The fact that this reduction is only observed in the birds with high methionine supplementation may shed some light on this issue. Previous studies showed that high consumption of diet rich in DHA increased methionine adenosyltransferase (MAT) activity and upregulated MAT mRNA expression in transmethylation metabolic pathway of methionine. The resultant increase in S-adenosylmethionine synthesis by MAT stimulates S-adenosylhomocysteine production, with the consequential upregulation of cystathionine *β*-synthase and cystathionine-*γ*-lyase, and as a result, removal of methionine permanently by converting it to cysteine [46–48]. Therefore, it may be speculated that the low dietary level of methionine impaired immune response and resulted in lower synthesis of IgG antibodies or perhaps thymus derived T-helper cells function [49, 50]. Pathologically also, we observed a reduction in bursa lesion score at 14 days after challenge in high methionine fed birds in our previous study [6]. 

## 5. Conclusion

Although there was no interaction between methionine × fish oil for plasma fatty acid profile, the significant interaction of cytokine response showed that a balance of moderate level of fish oil (2.5%) and methionine level (twice NRC recommendation) might enhance immune response in IBD challenged broiler chickens.

## Figures and Tables

**Table 1:** Ingredients and nutrients composition of experimental diets.

Ingredient (%)	Starter (1 to 21 d)						Finisher (22 to 42 d)					
	M_1_F_0_	M_2_F_0_	M_1_F_2.5_	M_2_F_2.5_	M_1_F_5.5_	M_2_F_5.5_	M_1_F_0_	M_2_F_0_	M_1_F_2.5_	M_2_F_2.5_	M_1_F_5.5_	M_2_F_5.5_
Corn	44.91	44.61	45.61	45.61	45.86	45.61	49.90	49.59	51.45	51.14	51.55	51.14
Soybean meal	43.85	43.60	43.73	43.18	43.48	43.18	38.67	38.47	38.43	38.23	38.33	38.23
Palm oil	6.58	6.58	0.00	0.00	0.00	0.00	7.31	7.31	0.00	0.00	0.00	0.00
Sunflower oil	0.00	0.00	3.50	3.50	0.50	0.50	0.00	0.00	3.50	3.50	0.50	0.50
Tuna oil	0.00	0.00	2.50	2.50	5.50	5.50	0.00	0.00	2.50	2.50	5.50	5.50
Dicalcium phosphate	1.91	1.91	1.91	1.91	1.91	1.91	1.77	1.77	1.77	1.77	1.77	1.77
Limestone	1.20	1.20	1.20	1.20	1.20	1.20	1.06	1.06	1.06	1.06	1.06	1.06
Salt	0.44	0.44	0.44	0.44	0.44	0.44	0.31	0.31	0.31	0.31	0.31	0.31
Vitamin premix^1^	0.30	0.30	0.30	0.30	0.30	0.30	0.30	0.30	0.30	0.30	0.30	0.30
Mineral premix^1^	0.30	0.30	0.30	0.30	0.30	0.30	0.30	0.30	0.30	0.30	0.30	0.30
DL-Methionine	0.25	0.80	0.25	0.80	0.25	0.80	0.23	0.74	0.23	0.74	0.23	0.74
Lysine	0.26	0.26	0.26	0.26	0.26	0.26	0.15	0.15	0.15	0.15	0.15	0.15
Calculated composition												
Crude protein	22.00	22.00	22.00	22.00	22.00	22.00	20.50	20.50	20.50	20.50	20.50	20.50
ME (Kcal/kg)	3080	3080	3080	3080	3080	3080	3150	3150	3150	3150	3150	3150
Available phosphorus	0.45	0.45	0.45	0.45	0.45	0.45	0.42	0.42	0.42	0.42	0.42	0.42
Calcium	1.00	1.00	1.00	1.00	1.00	1.00	0.90	0.90	0.90	0.90	0.90	0.90
Methionine	0.55	1.1	0.55	1.1	0.55	1.1	0.50	1.00	0.50	1.00	0.50	1.00
Lysine	1.20	1.20	1.20	1.20	1.20	1.20	1.00	1.00	1.00	1.00	1.00	1.00
Na	0.20	0.20	0.20	0.20	0.20	0.20	0.15	0.15	0.15	0.15	0.15	0.15
Analyzed composition												
Methionine	0.59	1.14	0.63	1.02	0.64	1.06	0.58	0.84	0.61	0.91	0.59	0.90
Lysine	1.26	1.28	1.19	1.29	1.22	1.19	1.11	1.01	1.05	1.13	1.09	1.10
Cysteine	0.42	0.34	0.40	0.38	0.47	0.37	0.35	0.34	0.26	0.35	0.40	0.32
DM	89.60	89.91	89.44	89.80	89.41	89.64	89.95	89.86	90.11	90.45	89.46	89.74
ASH	7.83	5.83	6.67	6.56	6.44	6.53	6.92	5.58	6.96	6.42	6.31	6.25
CP	22.43	21.76	22.45	22.87	21.79	21.85	21.20	21.07	20.40	21.11	21.15	20.88
EE	7.84	7.64	7.70	8.06	7.51	7.11	7.76	8.24	7.01	8.03	7.14	7.78
CF	3.50	3.11	3.64	2.21	3.44	3.68	4.32	4.44	2.95	3.17	3.47	2.50

^1^Supplied per kilogram of diet: vitamin A: 1,500 IU; cholecalciferol: 200 IU; vitamin E: 10 IU; riboflavin: 3.5 mg; pantothenic acid: 10 mg; niacin: 30 mg; cobalamin: 10 *μ*g; choline chloride: 1,000 mg; biotin: 0.15 mg; folic acid: 0.5 mg; thiamine: 1.5 mg; pyridoxine: 3.0 mg; iron: 80 mg; zinc: 40 mg; manganese, 60 mg; iodine: 0.18 mg; copper: 8 mg; selenium: 0.15 mg; BHT + EQ: 100 mg.

F_0_: 0% fish oil; F_2.5_: 2.5% fish oil; F_5.5_: 5.5% fish oil.

M_1_: methionine (NRC level); M_2_: methionine (2-fold of NRC).

**Table 2:** Fatty acid compositions of treatment diets.

	Fatty acid (%)	Starter (1–21 d)						Finisher (21–42 d)					
		M_1_F_0_	M_2_F_0_	M_1_F_2.5_	M_2_F_2.5_	M_1_F_5.5_	M_2_F_5.5_	M_1_F_0_	M_2_F_0_	M_1_F_2.5_	M_2_F_2.5_	M_1_F_5.5_	M_2_F_5.5_
Myristic	C14:0	0.81	0.73	1.05	1.05	2.61	2.75	0.84	1.25	2.20	1.30	2.44	2.62
Pentadecanoic	C15:0	0.04	0.05	0.33	0.33	0.86	0.91	0.27	0.10	0.59	0.38	0.80	0.83
Palmitic	C16:0	30.84	27.94	14.13	13.94	22.00	23.33	26.92	27.82	18.08	16.67	20.80	22.13
Palmitoleic	C16:1	0.17	0.18	1.27	1.27	3.09	3.21	0.20	0.22	2.20	1.37	2.82	3.02
Heptadecanoic	C17:0	0.12	0.1	0.80	0.81	1.97	2.01	0.09	0.05	1.28	0.81	1.80	1.84
Stearic	C18:0	3.69	3.33	4.34	4.34	5.82	6.31	3.09	3.05	4.52	4.47	5.54	5.92
Oleic	C18:1 *n-9 *	36.42	36.28	24.39	25.33	21.04	22.07	34.87	35.88	22.45	27.36	20.10	20.57
Linoleic	C18:2 *n-6 *	26.70	27.79	45.11	44.16	25.38	24.58	31.97	29.88	38.16	39.10	25.03	25.60
*α*-Linolenic.	C18:3 *n-3 *	0.61	0.59	0.21	0.22	0.27	0.27	0.73	0.67	0.34	0.39	0.43	0.48
Eicosapentaenoic (EPA)	C20:5 *n-3 *	ND	ND	0.06	0.05	0.10	0.21	ND	ND	2.29	2.02	1.96	1.96
Docosahexaenoic (DHA)	C22:6 *n-3 *	ND	ND	7.33	7.29	14.77	13.34	ND	ND	3.92	4.17	15.09	13.98

Total SFA		35.97	33.61	20.71	20.75	33.34	35.39	31.88	33.18	28.29	23.95	31.58	33.54
Total UFA		64.03	66.39	79.29	79.25	66.66	64.61	68.12	66.82	71.71	76.05	68.42	66.46
Total MUFA		36.72	38	26.05	26.99	25.05	26.23	35.43	36.27	25.33	29.16	23.76	24.50
Total PUFA *n-3 *		0.61	0.59	8.14	8.10	15.93	14.79	0.73	0.67	8.02	7.79	18.63	16.86
Total PUFA *n-6 *		26.70	27.79	45.11	44.16	25.38	24.58	31.97	29.88	38.16	39.10	25.03	25.60
UFA/SFA		1.78	2.07	3.83	3.82	2.00	1.83	2.14	2.02	2.78	3.18	2.17	1.99
PUFA/SFA		0.76	0.9	2.57	2.52	1.25	1.08	1.03	0.93	1.83	1.96	1.41	1.26
*n-6*/*n-3 *		43.77	47.10	5.54	5.45	1.59	1.66	43.79	44.59	4.76	5.02	1.34	1.52

SFA: saturated fatty acid; UFA: unsaturated fatty acid; MUFA: monounsaturated fatty acid; PUFA: polyunsaturated fatty acid.

Total saturated: sum of 8:0, 10:0, 12:0, 14:0, 15:0, 16:0, 17:0, and 18:0.

Total unsaturated: sum of 15:1, 16:1, 17:1, 18:1, 18:2 *n-6*, 18:3 *n-3*, 20:4 *n-6*, 20:5 *n-3*, 22:5 *n-3*, and 22:6 *n-3*.

Total MUFA: sum of 15:1, 16:1, 17:1, and 18:1.

Total PUFA *n-3*: sum of 18:3 *n-3*, 20:5 *n-3*, 22:5 *n-3*, and 22:6 *n-3*. Total PUFA *n-6*: sum of 18:2 *n-6* and 20:4 *n-6*.

F_0_: 0% fish oil; F_2.5_: 2.5% fish oil; F_5.5_: 5.5% fish oil.

M_1_: methionine (NRC level); M_2_: methionine (2 fold of NRC).

**Table 3:** Effect of fish oil and methionine supplementation on plasma fatty acids composition of 42-day-old broiler chickens (%).

	18:3 *n-3 *	20:5 *n-3 *	22:6 *n-3 *	18:2 *n-6 *	20:4 *n-6 *	Total *n-3* PUFA	Total *n-6* UFA	*n-6*/*n-3 *	PUFA/SFA
F_0_	0.63 ± 0.08	0.61 ± 0.24^c^	1.43 ± 0.51^c^	25.54 ± 0.86^a^	8.45 ± 0.44^a^	3.30 ± 0.74^c^	33.79 ± 0.83^a^	9.90 ± 0.39^a^	0.90 ± 0.042^b^
F_2.5_	0.82 ± 0.11	3.71 ± 0.31^b^	12.60 ± 0.67^b^	25.43 ± 1.04^a^	5.26 ± 0.55^b^	17.89 ± 0.92^b^	30.34 ± 1.07^a^	1.78 ± 0.50^b^	1.26 ± 0.54^a^
F_5.5_	0.92 ± 0.10	5.53 ± 0.28^a^	17.65 ± 0.61^a^	17.09 ± 0.94^b^	4.90 ± 0.51^b^	25.87 ± 0.81^a^	22.25 ± 0.98^b^	0.89 ± 0.46^b^	1.13 ± 0.49^a^
M_1_	0.59 ± 0.05	0.67 ± 0.16	1.37 ± 0.13	24.38 ± 1.16	7.75 ± 0.65	3.55 ± 0.25	32.36 ± 1.06	9.65 ± 0.60	0.85 ± 0.03
M_2_	0.68 ± 0.07	0.56 ± 0.29	1.30 ± 0.19	26.65 ± 1.40	8.74 ± 0.93	3.59 ± 0.27	35.73 ± 1.50	10.56 ± 0.96	0.95 ± 0.05

ANOVA (*P* value)									
M	0.65	0.056	0.57	0.97	0.79	0.37	0.66	0.71	0.29
F	0.09	0.0001	0.0001	0.0001	0.0001	0.0001	0.0001	0.0001	0.0001
M × F	0.62	0.09	0.81	0.24	0.95	0.38	0.51	0.96	0.47

^a–c^Means ± SEM within a column subgroup with no common letters differ at *P* < 0.05.

F_0_: 0% fish oil; F_2.5_: 2.5% fish oil; F_5.5_: 5.5% fish oil.

M_1_: methionine (NRC level); M_2_: methionine (2-fold of NRC).

**Table 4:** Effect of fish oil and methionine supplementation on blood parameters in broiler chickens challenged with IBD.

	Before challenge				7 d after challenge				14 d after challenge			
	WBC 10^9^/L	ALB (g/L)	TP (g/L)	GLU (g/L)	WBC 10^9^/L	ALB (g/L)	TP (g/L)	GLU (g/L)	WBC 10^9^/L	ALB (g/L)	TP (g/L)	GLU (g/L)
F_0_	17.6 ± 1.7^c^	11.7 ± 0.7	23.1 ± 1.1	11.4 ± 0.9^b^	40.4 ± 6.6^b^	10.3 ± 1.0	22.5 ± 1.2^b^	10.5 ± 1.2	49.0 ± 5.5	12.2 ± 1.2	28.1 ± 1.4	19.0 ± 1.7
F_2.5_	32.4 ± 1.7^b^	12.4 ± 0.7	21.0 ± 1.1	9.0 ± 0.8^b^	72.8 ± 7.4^b^	10.5 ± 1.0	23.3 ± 1.1^b^	10.8 ± 1.0	54.6 ± 6.2	14.9 ± 1.1	32.8 ± 1.2	19.4 ± 1.4
F_5.5_	40.2 ± 2.0^a^	10.0 ± 0.7	23.4 ± 1.1	15.9 ± 1.0^a^	80.4 ± 8.0^a^	12.4 ± 0.9	25.8 ± 1.1^a^	12.4 ± 0.9	54.5 ± 6.3	12.4 ± 1.2	29.3 ± 1.3	16.9 ± 1.6
M_1_	31.8 ± 1.5	10.3 ± 0.5	23.7 ± 0.9	12.7 ± 0.7	55.3 ± 6.2	11.8 ± 0.8	21.7 ± 0.9	9.3 ± 0.9^b^	48.4 ± 5.1	13.8 ± 0.9	29.0 ± 1.1	18.4 ± 1.3
M_2_	28.8 ± 1.5	11.9 ± 0.5	22.8 ± 0.9	12.3 ± 0.8	68.9 ± 5.8	10.5 ± 0.8	23.9 ± 0.9	13.8 ± 0.8^a^	59.2 ± 7.1	12.1 ± 1.0	29.5 ± 1.0	18.3 ± 1.2

ANOVA (*P* value)												
F	0.0001	0.07	0.32	0.003	0.002	0.22	0.04	0.01	0.96	0.27	0.98	0.85
M	0.15	0.09	0.49	0.99	0.18	0.45	0.12	0.26	0.35	0.24	0.12	0.44
F × M	0.14	0.97	0.97	0.11	0.27	0.61	0.52	0.36	0.54	0.51	0.30	0.16

^a–c^Means ± SEM within a column subgroup with no common letters differ at *P* < 0.05.

WBC: total white blood cell; TP: total protein; ALB: albumin; GLU: globulin.

F_0_: 0% fish oil; F_2.5_: 2.5% fish oil; F_5.5_: 5.5% fish oil.

M_1_: methionine (NRC level); M_2_: methionine (2-fold of NRC).

**Table 5:** Effect of fish oil and methionine supplementation on liver enzymes (U/L) in broiler chickens challenged with IBD.

	Before challenge		14 d after challenge	
	ALT	AST	ALT	AST
F_0_	15.5 ± 2.8	246 ± 10	3.8 ± 0.7	354 ± 30
F_2.5_	23.0 ± 3.5	260 ± 10	2.1 ± 0.5	361 ± 23
F_5.5_	21.2 ± 3.3	272 ± 10	3.6 ± 0.6	293 ± 27
M_1_	21.7 ± 2.1	272 ± 8	2.8 ± 0.6	337 ± 23
M_2_	18.8 ± 2.3	274 ± 8	3.6 ± 0.5	336 ± 20

ANOVA (*P* value)				
F	0.21	0.19	0.48	0.16
M	0.36	0.30	0.24	0.96
F × M	0.75	0.31	0.98	0.46

ALT: alanine aminotransferase; AST: aspartate aminotransferase.

F_0_: 0% fish oil; F_2.5_: 2.5% fish oil; F_5.5_: 5.5% fish oil.

M_1_: methionine (NRC level); M_2_: methionine (2-fold of NRC).

**Table 6:** Effect of fish oil and methionine supplementation on serum cholesterol and triglyceride levels (mmol/L) in broiler chickens challenged with IBD.

	Before challenge		14 d after challenge	
	CHOL	TRY	CHOL	TRY
F_0_	3.01 ± 0.12	0.70 ± 0.08	2.61 ± 0.15	0.67 ± 0.06
F_2.5_	3.14 ± 0.13	0.50 ± 0.08	2.78 ± 0.14	0.52 ± 0.06
F_5.5_	2.96 ± 0.12	0.51 ± 0.08	2.53 ± 0.15	0.49 ± 0.06
M_1_	2.96 ± 0.09	0.53 ± 0.06	2.70 ± 0.11	0.53 ± 0.04
M_2_	3.08 ± 0.10	0.59 ± 0.06	2.62 ± 0.12	0.59 ± 0.05

ANOVA (*P* value)				
F	0.17	0.50	0.71	0.10
M	0.76	0.14	0.63	0.37
F × M	0.001	0.54	0.09	0.32

^a–c^Means ± SEM within a column subgroup with no common letters differ at *P* < 0.05.

CHOL: cholesterol; TRY: triglyceride.

F_0_: 0% fish oil; F_2.5_: 2.5% fish oil; F_5.5_: 5.5% fish oil.

M_1_: methionine (NRC level); M_2_: methionine (2-fold of NRC).

**Table 7:** Effect of fish oil and methionine supplementation on serum IL-2 and IFN-*γ* levels (pg/mL) in broiler chickens challenged with IBD.

	IL-2			IFN-*γ*		
	Before challenge	2 d after challenge	7 d after challenge	Before challenge	2 d after challenge	7 d after challenge
F_0_	0.452 ± 0.008	0.365 ± 0.002	0.428 ± 0.002^b^	0.99 ± 0.07	1.38 ± 0.05	1.10 ± 0.04^a^
F_2.5_	0.442 ± 0.009	0.365 ± 0.003	0.423 ± 0.002^b^	0.98 ± 0.07	1.35 ± 0.05	0.87 ± 0.04^b^
F_5.5_	0.438 ± 0.008	0.374 ± 0.002	0.447 ± 0.002^a^	0.95 ± 0.06	1.39 ± 0.05	0.92 ± 0.04^b^
M_1_	0.440 ± 0.007	0.373 ± 0.002^a^	0.435 ± 0.002	1.06 ± 0.06	1.37 ± 0.04	0.93 ± 0.03^b^
M_2_	0.448 ± 0.007	0.361 ± 0.001^b^	0.432 ± 0.001	0.95 ± 0.05	1.37 ± 0.04	1.06 ± 0.03^a^

ANOVA (*P *value)						
F	0.57	0.051	0.006	0.88	0.76	0.002
M	0.41	0.008	0.54	0.49	1.00	0.04
F × M	0.18	0.014	0.10	0.26	0.14	0.01

^a–c^Means ± SEM within a column subgroup with no common letters differ at *P* < 0.05.

F_0_: 0% fish oil; F_2.5_: 2.5% fish oil; F_5.5_: 5.5% fish oil.

M_1_: methionine (NRC level); M_2_: methionine (2-fold of NRC).

**Table 8:** Influence of dietary treatments on serum IL-2 and IFN-*γ* levels (pg/mL) where fish oil × methionine interactions were significant in IBD challenged broiler chickens.

		IL-2 2 d after challenge			IFN-*γ* 7 d after challenge	
	F_0_	F_2.5_	F_5.5_	F_0_	F_2.5_	F_5.5_
M_1_	0.363 ± 0.003^b^	0.373 ± 0.003^abx^	0.385 ± 0.003^ax^	0.96 ± 0.05^y^	0.85 ± 0.05	0.97 ± 0.05
M_2_	0.367 ± 0.003	0.361 ± 0.003^y^	0.365 ± 0.003^y^	1.21 ± 0.05^ax^	0.90 ± 0.05^b^	0.93 ± 0.05^b^

^a–c^Means ± SEM within a row with no common letters differ at *P* < 0.05.

^x-y^Means ± SEM within a column with no common letters differ at *P* < 0.05.

F_0_: 0% fish oil; F_2.5_: 2.5% fish oil; F_5.5_: 5.5% fish oil.

M_1_: methionine (NRC level); M_2_: methionine (2-fold of NRC).
